# Association Between Bleeding Severity and Quality of Life in Adults with Immune Thrombocytopenia

**DOI:** 10.14789/ejmj.JMJ25-0055-OA

**Published:** 2026-02-10

**Authors:** MARVELOUS POPOOLA, SHARMEEN MALIK, KAUSHALENDRA MANI TRIPATHI, ASHOK KUMAR, IREM KARATAS, MUHAMMAD MUNEEB KHAN, BASIL KHALIL, SHOUGH ALHARATA, FARAH SALEH, SIDRA HARISS, MAJD ALDIN OUDEH, HIBA MANAF, KHUZAMI MOAIED RASHAD ODEH

**Affiliations:** 1 Department of Emergency Medicine, Calvary Adelaide Hospital, Adelaide, Australia; 1 Department of Emergency Medicine, Calvary Adelaide Hospital, Adelaide, Australia; 2 Department of Medicine, Holy Family Hospital, Rawalpindi, Pakistan; 2 Department of Medicine, Holy Family Hospital, Rawalpindi, Pakistan; 3 Department of Medicine, White River Medical Center - White River Health, Batesville, USA; 3 Department of Medicine, White River Medical Center - White River Health, Batesville, USA; 4 Department of Emergency Medicine, Lifecare Hospital, Abu Dhabi, UAE; 4 Department of Emergency Medicine, Lifecare Hospital, Abu Dhabi, UAE; 5 Department of Acute Medicine, Kingston and Richmond NHS Foundation Trust, London, United Kingdom; 5 Department of Acute Medicine, Kingston and Richmond NHS Foundation Trust, London, United Kingdom; 6 Department of Medicine, Hayatabad Medical Complex, Peshawar, Pakistan; 6 Department of Medicine, Hayatabad Medical Complex, Peshawar, Pakistan; 7 Department of Medicine, Istanbul Okan University, Istanbul, Turkey; 7 Department of Medicine, Istanbul Okan University, Istanbul, Turkey; 8 Department of General Practice, Gulf Medical University, Ajman, UAE; 8 Department of General Practice, Gulf Medical University, Ajman, UAE; 9 Department of General Practice, Thumbay University Hospital, Ajman, UAE; 9 Department of General Practice, Thumbay University Hospital, Ajman, UAE; 10 Department of Medicine, Odessa National Medical University, Odessa, Ukraine; 10 Department of Medicine, Odessa National Medical University, Odessa, Ukraine; 11 Department of Clinical Sciences, Thumbay University Hospital, Ajman, UAE; 11 Department of Clinical Sciences, Thumbay University Hospital, Ajman, UAE; 12 Department of General Practice, Ministry of Health Hospitals, Irbid, Jordan; 12 Department of General Practice, Ministry of Health Hospitals, Irbid, Jordan

**Keywords:** immune thrombocytopenia, bleeding severity, quality of life, WHOQOL-BREF, ISTH-BAT

## Abstract

**Objectives:**

Immune thrombocytopenia (ITP) is a disorder that is characterised by a low count of platelets and an increased risk of bleeding. It can cause symptoms which affect normal functioning and well-being. The study has discussed how bleeding severity is linked to the quality of life (QOL) of ITP patients in adulthood.

**Materials and Methods:**

A cross-sectional study was done to examine 300 patients with primary ITP admitted to tertiary care hospitals. Bleeding severity was measured with the help of the International Society on Thrombosis and Haemostasis Bleeding Assessment Tool (ISTH-BAT), and HRQoL was measured with the help of the WHOQOL-BREF. The data were analysed using Pearson's correlation, t-tests, ANOVA, and multiple linear regression.

**Results:**

Most participants (92.7%) were female, with a mean age of 49.8. Overall, HRQoL showed a significant negative correlation with bleeding severity (r = -0.42, p < 0.001). Women had higher bleeding scores (p < 0.001) and lower HRQoL scores (p = 0.017) than men. Worse bleeding and lower HRQoL were linked to older age (p < 0.001). Regression analysis revealed bleeding severity (β = -0.32, p = 0.001), female gender, old age, prolonged disease duration, frequent bleeding episodes, hospitalisation, and comorbidity as significant predictors of worse HRQoL.

**Conclusion:**

Adults with ITP have a worse quality of life in relation to greater bleeding severity. The holistic treatment that is concerned not only with the physical condition, but also with the emotional needs can contribute to the general welfare.

## Introduction

Immune thrombocytopenia (ITP) is an autoimmune disease characterised by isolated thrombocytopenia due to heightened platelet destruction and reduced platelet production. It can be primary or secondary and can happen in people of all ages, with bleeding as the most prevalent clinical manifestation^1, 2)^. ITP occurs at a rate of about 2-4 per 100,000 adults^3)^.

The bleeding in ITP can range from mildly bruised to life-threatening intracranial haemorrhage. There is a sharp rise in the risk of bleeding with very low platelet counts, especially below 20 × 10^9^/L, and severe or mucosal bleeding is associated with extremely low levels and the use of anticoagulant drugs^4, 5)^.

ITP causes significant loss in health-related quality of life (HRQoL), whereby the patients are usually fatigued, have a fear of bleeding and experience social or occupational restrictions. Recent research highlights the importance of patient-reported outcomes^6)^. Fatigue was strongly associated with diminished HRQoL in the physical and mental domains^7)^. In general, ITP and its management have significant influences on patients' HRQoL, particularly in chronic cases. Factors such as low platelet count, anaemia, bleeding severity, and various treatments can deteriorate QOL, underscoring the importance of holistic care aimed at enhancing well-being^8, 9)^.

Given that there is limited literature on the developing world, particularly South Asian clinical practice, it would be necessary to find out the effect of bleeding symptoms on QoL among adults with ITP. The study aims to establish the association between HRQoL and bleeding severity in adults with ITP. Identifying such a connection may help clinicians develop more comprehensive management strategies to address the disease's psychosocial and physical aspects.

## Materials and Methods

### Study design and setting

The study is a cross-sectional observational study of patients with ITP who visited haematology outpatient clinics at tertiary care hospitals. Data collection was conducted over 7 months following institutional review board approval.

### Study population and sampling

The study population consisted of 300 patients with confirmed primary ITP. The sample size was calculated using the correlation studies formula, with a 95% confidence level and a 5% margin of error. Participants were recruited through convenience sampling.

The inclusion criteria were adults aged 18 years or older with a history of ITP for at least 3 months. Patients with secondary thrombocytopenia, comorbid hematologic or severe psychiatric illness were excluded. Informed consent was obtained in writing from all participants before data collection.

### Data collection tools

#### Demographic and clinical proforma

Demographic and clinical factors, including age, gender, education, length of illness, current treatment, and comorbidities, were recorded using a structured proforma.

#### ISTH-BAT (International society on thrombosis and haemostasis - bleeding assessment tool)

The severity of bleeding was assessed by the ISTH-BAT, created by the Scientific and Standardisation Committee (SSC) of the International Society on Thrombosis and Haemostasis in 2010. The ISTH- BAT consists of 14 types of bleeding, including epistaxis, cutaneous bleeding, oral cavity bleeding, gastrointestinal bleeding, and surgical bleeding, with a score of 0 to 4 (0 = no bleeding, 1-2 = mild/moderate, 3 4 = severe). The cumulative score is the bleeding phenotype of the individual. The highest possible score is 56, and the higher the score, the more severe the tendency toward bleeding^10)^.

#### WHOQOL-BREF (World health organisation quality of life - BREF)

The WHOQOL-BREF, developed by the World Health Organisation Quality of Life group in 1996, was used to measure QOL. The questionnaire consists of 26 questions and covers four areas: physical health, psychological health, social relationships, and environment. Individual items are rated using a 5-point Likert rating scale (1 = very poor, 5 = very good). Raw domain scores are transformed to a 0-100 scale, with higher scores indicating a better quality of life^11)^.

### Procedure

Face-to-face interviews with trained researchers were conducted in a private setting where data were collected and confidentiality was maintained. All questionnaires were completed during a single visit, and each session lasted about 20-25 minutes. The records were collected anonymously and stored in secure locations.

### Data analysis

The statistical analysis was conducted using the Statistical Package for the Social Sciences (SPSS) version 26.0. A descriptive summary of the participants' characteristics was presented using frequencies and percentages. Pearson's correlation and independent t-tests were used to examine the relationship between bleeding severity (ISTH- BAT scores) and quality of life (WHOQOL-BREF domains). A p-value less than 0.05 was taken to be statistically significant.

### Ethical considerations

The Holy Family Hospital, Rawalpindi, ethics committee granted ethical permission (HFH/ERC- 2025-A/09-147). Participation was voluntary, and all participants provided informed written consent before enrolment. The research process was done in a highly confidential and anonymous manner.

### Demographic characteristics of participants (N = 300)

A total of 300 participants were included in the study, as indicated in Table 1, most of them female (92.7%), with the 50-59 age group being the most significant (26.7%). The vast majority of participants were divorced (37.7%) or married (33.3%), and had secondary-level education or higher (58.6% combined). Regarding employment, 32.7% were students, and 28.0% were not employed. Most of them had 1-3 years of ITP diagnosis (35.3%). The most prevalent treatments currently were IVIG (28.3) and corticosteroids (26.0), and 14.7% had no treatment. More than half experienced bleeding in at least one hospitalisation (53.3%), and 36.0% had occasional bleeding episodes within the last six months. The most prevalent comorbidities were hypertension (37.7%) and diabetes (32.7%), whereas 16.0% did not use any other chronic illness.

**Table 1:** Demographic characteristics of participants (N = 300)

Variable	f (N)	%		Variable	f (N)	%
Age				Duration since ITP diagnosis		
18-29 years	40	13.3		Less than 6 months	40	13.3
30-39 years	55	18.3		6-12 months	77	25.7
40-49 years	70	23.3		1-3 years	106	35.3
50-59 years	80	26.7		3-5 years	58	19.3
60 years or above	55	18.3		More than 5 years	19	6.3
Gender				Current treatment		
Male	22	7.3		No treatment/watchful waiting	44	14.7
Female	278	92.7		Corticosteroids	78	26.0
Marital status				IVIG (Intravenous Immuno-globulin)	85	28.3
Single	45	15.0		Thrombopoietin receptor agonists (e.g., eltrombopag, romiplostim)	68	22.7
Married	100	33.3				
Divorced	113	37.7		Splenectomy	25	8.3
Widowed	42	14.0		Frequency of bleeding episodes (past 6 months)		
Educational level				None	108	36.0
No formal education	12	4.0		Occasional (1-2 times)	108	36.0
Primary	49	16.3		Moderate (3-5 times)	58	19.3
Secondary	87	29.0		Frequent (more than 5 times)	26	8.7
Undergraduate	88	29.3		Any hospitalisation due to bleeding?		
Graduate or above	64	21.3		Yes	160	53.3
Employment status				No	140	46.7
Student	98	32.7		Presence of other chronic illness		
Employed	21	7.0		None	48	16.0
Home-maker	70	23.3		Hypertension	113	37.7
Unemployed	84	28.0		Diabetes	98	32.7
Retired	27	9.0		Heart disease	41	13.7

Note. f = frequency, % = percentage; Values are presented as N (%), N = 300; No statistical comparisons were performed for demographic variables in this table.

### Correlation between bleeding severity and quality of life

As indicated in Table 2, there was a strong negative relationship between bleeding severity (ISTH- BAT) and overall quality of life (WHOQOL-BREF) scores (r = -0.420, p < 0.001). This shows that the participants who had more severe bleeding had a lower quality of life in physical, psychological, social, and environmental areas.

**Table 2:** Correlation Between Bleeding Severity (ISTH-BAT) and quality of life (WHOQOL-BREF) among adults with immune thrombocytopenia (N = 300)

Variables	r	t(df)	p
Total ISTH-BAT	-	-	-
Total WHOQOL-BREF	-0.420**	-7.99 (298)	<0.001**

Note. Values represent Pearson correlation coefficients (r) between continuous variables; N = 300; p < 0.001 (2-tailed) was considered statistically significant and is denoted with double asterisks (**).

### Gender differences in bleeding severity and quality of life

Table 3 shows that quality of life and bleeding severity differed significantly by gender. Females (225.0 ± 10.5) scored higher than males (235.5 ± 12.5) in terms of mean bleeding severity scores (ISTH-BAT: 235.5 ± 12.5), and males had milder bleeding. Conversely, the overall QOL was observed to be poorer in the case of females (WHOQOL-BREF: 75.5 ± 6.5) when compared with that of males (72.0 ± 8.0; t = 2.42, p = 0.017, Cohen d = 0.46).

**Table 3:** Gender differences in bleeding severity and quality of life among patients (N = 300)

Variables	Male (N=22; 7.3%); (M ± SD)	Female (N=278; 92.7%); (M ± SD)	t	p	95% CI LL	95% CI UL	Cohen's *d*
Total ISTH-BAT	225.00 ± 10.50	235.50 ± 12.50	-4.48	<0.001***	-15.60	-5.40	0.87
Total WHOQOL-BREF	75.50 ± 6.50	72.00 ± 8.00	2.42	0.017*	0.65	6.35	0.46

Note. Values are presented as Mean ± Standard Deviation; Independent samples t-tests were conducted to compare participants of both male and female groups; Group sizes are shown as N (%); A p-value < 0.001 was considered statistically significant, N = 300.

### Age-related differences in bleeding severity and quality of life

According to Table 4, participants showed significant age-related differences in quality of life and bleeding severity. Bleeding (ISTH-BAT) was more severe in the 60+ years group (240.0 ± 12.0) compared to the 18-29 years age group (220.0 ± 8.0) (F = 12.30, p = 0.001, 0.142). Conversely, the overall QOL (WHOQOL-BREF) deteriorated with age, ranging from 76.0 ± 6.0 to 68.0 ± 8.5 (F = 6.30, p < 0.001, η 2 = 0.079), indicating that older respondents reported more bleeding and lower perceived well-being.

**Table 4:** Age-related differences in bleeding severity and quality of life among patients (N = 300)

Variables	18-29 years (N=40;13.3); (M ± SD)	30-39 years (N=55;18.3); (M ± SD)	40-49 years (N=70;23.3); (M ± SD)	50-59 years (N=80;26.7); (M ± SD)	60+ years (N=55;18.3); (M ± SD)	F(4,295)	p	η^2^
Total International Society on Throm-bosis and Haemo-stasis - Bleeding Assessment Tool (ISTH-BAT)	220.0 ± 8.0	225.0 ± 9.0	230.0 ± 10.0	235.0 ± 11.0	240.0 ± 12.0	12.30	<0.001***	0.142
Total World Health Organization Quality of Life (WHOQOL-BREF)	76.0 ± 6.0	74.0 ± 7.0	72.0 ± 7.5	70.0 ± 8.0	68.0 ± 8.5	6.30	<0.001***	0.079

Note. Data are presented as Mean ± Standard Deviation (M ± SD); Group sizes are shown as N (%). One-way ANOVA was conducted to examine the effect. All comparisons were significant at p < 0.001; η^2^ represents the partial eta-squared effect size.

### Multiple regression predicting QOL from bleeding severity and clinical factors

In participants with ITP, as indicated in Table 5, multiple linear regression analysis shows several significant predictors of quality of life (WHOQOL-BREF). Lower quality of life was linked to higher bleeding severity (ISTH-BAT) (β = 0.320 = -0.001). Also, female gender (β = -0.110, p = 0.006), older age (β = -0.180, p < 0.001), longer disease time (β = -0.120, p = 0.002), more frequent bleeding episodes (β = -0.200, p < 0.001), bleeding hospitalizations (β = -0.130, p = 0.003), and other chronic illnesses (β = -0.110, p = 0.003) were all predictors of lower quality of life. All these aspects indicate the multifactorial influence of clinical and demographic factors on patients' well-being.

**Table 5:** Multiple Linear Regression Predicting QOL (WHOQOL-BREF) from bleeding severity, demographics, and clinical factors (N = 300)

Model	B	SE	β	t	p	95% CI Lower LL	95% CI Upper UL
(Constant)	85.000	5.000	-	17.00	<0.001***	75.200	94.800
Total ISTH-BAT	-0.230	0.035	-0.320	-6.57	<0.001***	-0.299	-0.161
Gender (Male=1, Female=2)	-2.500	0.900	-0.110	-2.78	0.006**	-4.268	-0.732
Age (years)	-0.150	0.040	-0.180	-3.75	<0.001***	-0.229	-0.071
Duration since ITP diagnosis (years)	-0.250	0.080	-0.120	-3.13	0.002**	-0.407	-0.093
Frequency of bleeding episodes (past 6 months)	-0.800	0.200	-0.200	-4.00	<0.001***	-1.196	-0.404
Any hospitalisation due to bleeding?	-1.800	0.600	-0.130	-3.00	0.003**	-2.984	-0.616
Presence of other chronic illness	-1.500	0.500	-0.110	-3.00	0.003**	-2.480	-0.520

Note. Values include unstandardized coefficients (B), 95% confidence intervals (CI), standard errors (SE), standardised beta coefficients (β), and p-values; **= p < 0.01, ***=p < 0.001 were considered statistically significant; N = 300.

### Regression coefficients predicting quality of life (WHOQOL-BREF)

**Figure 1:**
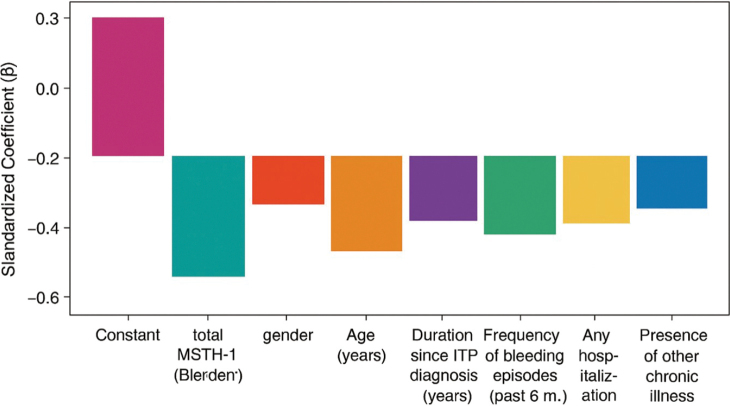
Bar chart for regression predicting quality of life (WHOQOL-BREF) (N = 300)

Figure 1 shows the regression coefficients (B) standardised to predict QOL, as measured by the WHOQOL-BREF, for 300 patients with immune thrombocytopenia (ITP). The chart demonstrates a significant negative correlation between higher total ISTH-BAT (bleeding) scores and quality of life, suggesting that more severe bleeding was strongly and negatively associated with patients' well-being. Other independent variables, such as gender, age, years since ITP diagnosis, the number of bleeding episodes in the last six months, hospitalisation history, and the presence of other chronic conditions, also correlated negatively with quality of life, but the effects were relatively weak. On the whole, bleeding severity turned out to be the most significant predictor of poor quality of life in patients with ITP.

## Discussion

The current study investigated the association between bleeding severity and QoL in adults with ITP in a South Asian clinical setting. Our results showed a significant negative correlation between bleeding severity and QoL, indicating that patients with more severe bleeding have lower overall well-being. This is in line with previous research on bleeding symptoms and fear of bleeding as determinants of fatigue, limitations in activity, and social and occupational functioning^12)^.

There were also gender-specific differences because the female patients noted a higher level of bleeding and poorer QoL than their male counterparts. These findings are consistent with the previous research that has already determined that women with bleeding disorders were more likely to bleed in comparison with menstruation, sex and childbirth and were more fatigued and emotionally distressed^13, 14)^. This shows the importance of gender sensitive management in ITP. This highlights the significance of gender sensitive management strategies in ITP.

Age was another factor which influenced the degree of bleeding and QoL. The ISTH-bat scores of the older patients were poorer, and their QoL was lower, in accordance with available data showing that older age predisposes older people to severe bleeding and other associated issues^15, 16)^. It is possible to correlate age-related deterioration in quality of life with accumulating disease burden, comorbid conditions, and treatment-related problems.

The other significant finding of our study is that, according to regression analysis, bleeding severity, older age, and female gender are potent predictors of lower QoL, consistent with the literature ^12, 14, 16)^.

Likewise, lower QoL was associated with increased disease duration and repeated bleeding events, which are manifestations of physical discomfort, anxiety, and lifestyle restriction related to chronic exposure to ITP symptoms and treatment^17, 18)^.

The other serious predictor of worse QoL was the extensive bleeding that led to hospitalisation, likely due to the physical, psychological and social turmoil such events created. Further, comorbid chronic illnesses augmented the health burden and further reduced the QoL^16, 19)^.

All in all, the findings demonstrate that the intensity of bleeding, gender, age, disease duration, hospitalisation, and comorbidities influence QoL in ITP patients. Knowledge of such factors may guide clinicians in providing personalised interventions to mitigate the impact of diseases and improve patients' well-being.

### Study constraints

Several limitations must be noted. First, a cross- sectional design does not allow for causal inference between bleeding severity and QOL; longitudinal research is required to determine the direction of causation. Second, convenience sampling was used, limiting generalizability to community settings or other rural populations and potentially introducing selection bias. Third, self-reported instruments were used in data collection and can be subject to recall or response bias. Fourth, the patients were not stratified by treatment phase (newly diagnosed, persistent, or chronic ITP), which could have affected bleeding profiles and QoL outcomes. Finally, the other psychosocial variables, such as social support and coping techniques, were not quantified but may have mediated the identified relationships.

## Conclusion

The research indicates that bleeding severity is closely linked to QOL in adults with ITP. Patients who have more severe bleeding, repeated hospitalisations, and comorbidities are more physically and psychologically impaired. These challenges are further aggravated by female gender, old age, and prolonged disease period. The findings indicate that clinicians must adopt a multidimensional approach to treating ITP, extending beyond platelet count to consider patients' mental, social, and emotional health. Early detection of bleeding symptoms and active monitoring of HRQoL enable the implementation of a personalised intervention to improve overall patient outcomes.

## Author contributions

MP conceived and designed the study and supervised the overall project. SM contributed to the study design, clinical assessments, and interpretation of WHOQOL-BREF outcomes. KMT assisted in data acquisition and clinical interpretation. AK contributed to patient recruitment and hospital data collection, while IK supported data collection and clinical analysis. MMK was responsible for statistical analysis and drafting of results. BK participated in the literature review and manuscript drafting. SA assisted in data entry and quality checks. FS contributed to the study methodology and revision of the manuscript. SH assisted with data management and formatting of tables and figures. MO participated in the literature review, proofreading, and critical revision of the manuscript. HM contributed to clinical assessments and manuscript editing. KO assisted in data coordination and final manuscript formatting. All authors read and approved the final manuscript.

## Conflicts of interest statement

The authors declare that there are no conflicts of interest.
